# Adverse drug reaction reporting with the Med Safety app in Uganda: a cluster-randomised, controlled trial

**DOI:** 10.1016/S2214-109X(25)00299-2

**Published:** 2025-09-17

**Authors:** Ronald Kiguba, Helen B Ndagije, Norah Mwebaza, Ronald Ssenyonga, Lilian Giibwa, Gerald Isabirye, Jonathan Owiny, Victoria Nambasa, Ismail Ntale, Joanitah Atuhaire, Douglas Mwesigwa, Julius Mayengo, David Walusimbi, Ian Mugisa, Cordelia Katureebe, Kendal Harrison, Charles Karamagi, Munir Pirmohamed

**Affiliations:** a Department of Pharmacology and Therapeutics, School of Biomedical Sciences, College of Health Sciences, Makerere University, Kampala, Uganda; b National Pharmacovigilance Centre, National Drug Authority, Kampala, Uganda; c Department of Epidemiology and Biostatistics, School of Public Health, College of Health Sciences, Makerere University, Kampala, Uganda; d African Union Development Agency, Johannesburg, South Africa; e AIDS Control Programme, Ministry of Health, Kampala, Uganda; f Medicines and Healthcare products Regulatory Agency, London, UK; g Clinical Epidemiology Unit, School of Medicine, College of Health Sciences, Makerere University, Kampala, Uganda; h Centre for Drug Safety Science and Wolfson Centre for Personalised Medicine, Institute of Systems, Molecular and Integrative Biology (ISMIB), University of Liverpool, Liverpool, UK; i Department of Public Health, Faculty of Health Sciences, Muni University, Kampala, Uganda

## Abstract

**Background:**

The massive roll-out of new and repurposed medicines in low-income and middle-income countries (LMICs) highlights the need for more efficient pharmacovigilance systems, including use of digital technologies. We assessed the effectiveness of the Med Safety app in improving suspected adverse drug reaction (ADR) reporting by health-care workers to Uganda's National Pharmacovigilance Centre.

**Methods:**

This was a pragmatic, multicentre, open-label, cluster-randomised, controlled trial undertaken at health facilities (clusters), providing dolutegravir-based combination antiretroviral therapy in Uganda. Clusters were randomly assigned (1:1) to the intervention group or control group using a computer-generated simple randomisation sequence. In the intervention group, pharmacists with expertise in pharmacovigilance delivered 2 h of face-to-face training to health-care workers in clusters, regardless of their smartphone ownership, in Med Safety and traditional ADR reporting methods. The control group received the same training as the intervention group except for Med Safety training. The primary outcome was the cluster-level ADR reporting rate at the end of follow-up and was analysed in all sites that received the allocated intervention. The trial is registered with the Pan African Clinical Trials Registry (PACTR202009822379650) and is completed.

**Findings:**

Between Aug 11, 2020 and Nov 1, 2022, 382 clusters were randomly assigned and 367 received the allocated intervention and were included in the primary outcome analysis (184 in the intervention group and 183 in the control group), with 2464 health-care workers (1211 in the intervention group and 1253 in the control group). The follow-up time for the included clusters was variable and was median 37·8 months (IQR 34·2–39·8). In the primary analysis, the intervention group had a higher mean overall ADR reporting rate of 10·6 (SD 17·4) reports per 100 000 person-months versus 5·9 (17·9) in the control group (incidence rate ratio 1·73 [95% CI 1·26–2·37]; p=0·001).

**Interpretation:**

Med Safety increased ADR reporting rates among health-care workers in Uganda. Integrating digital technologies into pharmacovigilance systems could strengthen drug-safety monitoring in Uganda and other LMICs.

**Funding:**

UK Medical Research Council and the Foreign Commonwealth and Development Office; Makerere University Research & Innovations Fund; and Ugandan National Drug Authority.

## Introduction

Spontaneous reporting of suspected adverse drug reactions (ADRs) is the cornerstone of pharmacovigilance systems; however, under-reporting represents a major weakness.^1^ Health-care workers remain the main contributors to spontaneous ADR reporting systems. Effective ADR reporting, however, is hindered by the low availability and cumbersome nature of paper forms, limited access to web forms, uncertainty about reporting channels, and inadequate feedback given to reporters.^1^ Therefore, the development of digital technologies, such as smartphone apps, to promote ADR reporting is necessary, particularly in low-income and middle-income countries (LMICs) where the ADR reporting rate is very low.^2^, ^3^ Ideally, these digital tools should integrate with existing pharmacovigilance systems.^4^

In 2018, Uganda rolled out dolutegravir as the preferred drug for first-line and second-line combination antiretroviral therapy. Nearly 350 000 people living with HIV were transitioned onto or initiated dolutegravir during the first 12 months since it became available in Uganda.^5^ In 2019, Uganda simultaneously scaled up isoniazid preventive therapy, hereafter isoniazid, for the prevention of, and reduction of mortality from, tuberculosis among people with HIV.^5^ During the 100-day isoniazid scale-up campaign from July to September, 2019, the programme enrolled over 343 600 people with HIV, exceeding the target of 304 400.^5^, ^6^

The massive roll-out of dolutegravir and isoniazid in Uganda highlighted the need for more efficient national drug-safety monitoring systems, including the use of digital technologies.^5^, ^7^ Thus, the Med Safety app was introduced to support existing methods of ADR reporting.^8^ Med Safety was derived from the prototype mobile app initially developed by the WEB-Recognising Adverse Drug Reactions (WEB-RADR) project, the prototype being adopted between 2015 and 2016 by the UK's Medicines and Healthcare products Regulatory Agency, the Netherlands’ Lareb and Croatia's HALMED.^2^, ^9^ In 2017, the original version of Med Safety was introduced in Africa (Burkina Faso and Zambia) by Medicines and Healthcare products Regulatory Agency in partnership with the WEB-RADR project and WHO.^2^, ^3^, ^10^ Med Safety was launched in Uganda in 2020. Its potential to promote digital pharmacovigilance in LMICs had not previously been investigated,^8^ and we therefore conducted a qualitative study to evaluate the acceptability and feasibility of introducing Med Safety to health-care workers. This showed that health-care workers were willing to use the app to report; however, more training and practice were needed to improve uptake.^11^

**Research in context**

**Evidence before this study**

On Dec 1, 2022, we searched PubMed, Embase, and Web of Science with no language restrictions for publications from inception to Dec 1, 2022, using the search terms: (((Mobile Applications) OR (Smartphone Applications) OR (Mobile Apps) OR (Smartphone Apps) OR (Mobile Technology) OR (Mobile Health Technology) OR (Mobile Health Applications) OR (Mobile Health Apps) OR (Web Applications) OR (Web Platforms)) OR (Electronic Health Records)) AND ((Pharmacovigilance) OR (Adverse Event Reporting) OR (Adverse Drug Reaction Reporting) OR (Suspected Adverse Drug Reaction Reporting) OR (Drug Monitoring) OR (Drug Safety Monitoring) OR (Drug Surveillance) OR (Drug Safety Reporting) OR (Medication Monitoring) OR (Medication Side Effect*) OR (ADR Investigation) OR (ADR Management) OR (ADR Response) OR (Drug-Related Side Effect*) OR (Drug-Related Adverse Reaction*) OR (Drug Side Effect*) OR (Side Effect*) OR (Adverse Drug Reaction*) OR (Adverse Reaction*) OR (Adverse Drug Event*) OR (Adverse Event*))) AND (((health worker*) OR (health care worker*) OR (healthcare worker*) OR (health care professional*) OR (healthcare professional*) OR (health professional*) OR (doctor*) OR (physician*) OR (nurse*) OR (pharmacist*) OR (clinical officer*) OR (medical officer*) OR (midwife*) OR (community health worker*) OR (community health care worker*) OR (community healthcare worker*))). We identified no cluster-randomised controlled trials or systematic reviews on smartphone app-based reporting of suspected adverse drug reactions (ADRs) by health-care workers in low-income and middle-income countries (LMICs). Our search results showed that other electronic reporting systems—such as web-based platforms and electronic health records—improved ADR reporting by health-care workers, with increases ranging from 1·5 to 5-times. A systematic review and meta-analysis of electronic reporting systems showed a doubling of ADR reporting, although the quality of evidence was low and LMICs were under-represented. We updated the search on June 30, 2025, and found one cluster-randomised controlled trial on mobile app-based ADR reporting by health-care workers in an LMIC. This cluster trial, conducted in Laos had a small sample size of one hospital in the intervention group with 31 health-care workers and one hospital in the control group with 34 health-care workers; and a follow-up period of only 4 months. The reported incidence rate ratio was 10·50 (95% CI 3·24–53·97; p<0·001). Importantly, the analysis did not adjust for clustering, limiting the validity of the findings given the clustered study design.

**Added value of this study**

To our knowledge, this is the first robust pragmatic cluster-randomised controlled trial to test the impact of a mobile app, Med Safety, on ADR reporting by health-care workers, with the evidence generated from an LMIC setting. We found that Med Safety increased the rate of ADR reporting. The positive impact of Med Safety on ADR reporting by health-care workers highlights the value of smartphone apps in strengthening pharmacovigilance systems in Uganda. Med Safety's success could potentially be replicated in other LMICs.

**Implications of all the available evidence**

These findings support integrating digital technologies such as Med Safety into pharmacovigilance systems in Uganda and other LMICs, which could strengthen drug safety monitoring and contribute to more robust, data-driven, regulatory decision making in these settings.

There is scarce literature on the impact of mobile apps on ADR reporting in LMICs. We therefore designed a large, pragmatic, cluster-randomised, controlled trial to assess the effectiveness of Med Safety in improving the rate of ADR reporting by health-care workers to Uganda's National Pharmacovigilance Centre (NPC).^8^ We envisaged Med Safety to be more efficient, user-friendly, and readily accessible to reduce barriers to timely ADR reporting, unlike traditional ADR reporting methods. We aimed to test the hypothesis that introducing Med Safety to complement existing pharmacovigilance methods would increase the rate of ADR reporting to NPC by health-care workers by at least 25% versus using existing pharmacovigilance methods.

## Methods

### Study design and participants

This was a pragmatic, multicentre, open-label, cluster-randomised, controlled trial undertaken at 367 (96%) of 382 high-volume health facilities providing dolutegravir-based combination antiretroviral therapy that represented 20% of all accredited HIV clinics nationwide and served 80% of people with HIV receiving dolutegravir-based regimens in Uganda.^12^ In Uganda's health-care system, health facilities differ in the level of health-care services provided. Level 1 is village health teams and has no physical site location (community health workers provide health services directly to the community), level 2 health centres provide primary health care, level 3s offer both primary and some secondary health care, level 4s deliver primary and secondary health care, and hospitals provide secondary and tertiary health care.^13^ Standalone specialised HIV clinics (not categorised within the level system) provide tertiary care and are research centres of excellence. To ensure uniform HIV health care across all levels of clinics nationwide, standardised HIV training is provided to all health-care workers.

All health-care workers, including physicians, medical officers, pharmacists, nurses and midwives, clinical officers, pharmacy technicians, and community health workers, who cared for people with HIV receiving dolutegravir-based regimens at the enrolled health facilities (of any level), hereafter called clusters, were eligible. We obtained written informed consent from the enrolled health-care workers.

The trial was registered with the Pan African Clinical Trials Registry (PACTR202009822379650) and is completed. The protocol has been published previously.^8^ Ethical approval was obtained from the School of Biomedical Sciences Research and Ethics Committee, Makerere University (SBS-REC-720), and Uganda National Council for Science and Technology (HS1366ES). Administrative clearance was obtained from the health facilities. An independent Data Safety Monitoring Board (DSMB) oversaw the trial.

### Randomisation and masking

The unit of randomisation was a cluster, a pre-screened health facility, consisting of health-care workers who care for people with HIV receiving dolutegravir-based regimens. Using health facilities as clusters aimed to minimise contamination, duplicate reports, and organisational challenges, and provided valid population denominators of people with HIV for analysing the outcomes. An independent biostatistician from the Clinical Epidemiology Unit, Makerere University in Kampala, Uganda, who did not participate in the subsequent data analysis, generated the simple randomisation sequence. The number of clusters was considered adequately large for effective balance between study groups by simple randomisation. Using the simple randomisation sequence, research assistants (part of the research team of the study) randomly assigned (1:1) clusters to the intervention or control groups.

The trial masked the allocation of clusters to pharmacovigilance assessors at NPC and the biostatistician who analysed the data. However, research assistants and health-care workers were not masked due to the nature of the intervention. To minimise unmasking, we informed research assistants not to share allocation status with masked groups. It was not possible to stop health-care workers from transferring information across study groups. Therefore, sample size planning allowed for 30% contamination,^14^ although only 8% of control sites reported with Med Safety.

### Procedures

Research assistants were responsible for collecting high-quality survey data and received initial and ongoing training on key concepts in pharmacovigilance, Good Clinical Practice, Human Subject Protection, randomisation, risk profiles of combination antiretroviral therapy, and tuberculosis preventive therapy. Strict enrolment protocols with regular supervision were instituted.

Enrolled health-care workers in each cluster completed an interviewer-administered electronic questionnaire (appendix pp 3–6) using Open Data Kit software to capture the health-care workers’ baseline characteristics. The in-charge health-care worker (administrative heads of clinics) in each cluster provided the cluster's baseline characteristics.

In the intervention group, the research team introduced Med Safety to health-care workers regardless of their ownership of a smartphone. Pharmacists with expertise in pharmacovigilance delivered face-to-face training to health-care workers using a training schedule standardised across all health facilities. Interested health-care workers were invited and assisted to install Med Safety on their own smartphone, and trained to use it to report all ADRs, with emphasis on dolutegravir-related and isoniazid-related ADRs. Enrolled health-care workers were also trained to use traditional ADR reporting methods, including paper forms and web forms. Posters and brochures about ADR reporting were distributed, and monthly reminders about ADR reporting sent to health-care workers for the first 6 months via WhatsApp and mobile phone short messages.^8^ The entire training session took approximately 2 h in the intervention group.

In the control group, pharmacists with expertise in pharmacovigilance trained health-care workers to use traditional methods of ADR reporting with the training being identical to that in the intervention group (with emphasis on dolutegravir-related and isoniazid-related ADRs), except that Med Safety was not introduced to health-care workers in the control group. Posters, brochures, and reminders were used, consistent with the intervention group.^8^

In the intervention group, health-care workers with smartphones could use Med Safety to submit ADR reports directly to NPC. The duration of follow-up was initially planned for 30 months of follow-up per health facility but was variable for the different study sites. Med Safety is hosted by Uganda's National Drug Regulatory Authority where the NPC is situated. Med Safety captures details on patient sociodemographics, suspect medicine, concurrent medicines, suspected ADRs, and medical history. Paper forms and the web form links were distributed in all enrolled clusters. Pharmacovigilance focal people and drug-regulatory officers collected completed paper forms and submitted them to NPC for entry into the national pharmacovigilance database and health-care workers used the web form to submit ADR reports directly to the national pharmacovigilance database. At baseline, we documented the reasons why some health-care workers in the intervention group did not install Med Safety.

A pharmacovigilance database quality control officer (member of the research team and NPC) was trained and empowered to embed data (all data from Med Safety, paper forms, and web forms) quality management into the national pharmacovigilance database.

### Outcomes

The primary outcome was the cluster-level reporting rate of ADRs submitted by health-care workers to Uganda's NPC. The secondary outcomes were the cluster-level reporting rates of dolutegravir-specific ADRs, isoniazid-related ADRs, and of serious and non-serious ADRs. These rates were expressed as the number of ADR reports per 100 000 dolutegravir person-months of observation. We excluded analysis of app downloads, ADR causality, and ADR outcomes, as app download data could not be disaggregated by cluster, and ADR causality and ADR outcome data lacked consistency in the national pharmacovigilance database. Economic evaluation data will be reported separately.

### Statistical analysis

The unit of analysis was a cluster in keeping with the cluster-randomised design. The study used the Hayes and Bennet method to estimate the number of clusters required per group for an effect size of 25%,^15^ assuming a mean of 1·0 ADR report (SD 1·2) per 100 000 dolutegravir person-months of observation. This ADR reporting rate was based on 1-year data from the national pharmacovigilance database.^8^ Sample size estimation employed the lower reporting rate of isoniazid-related ADRs (versus the higher rate of dolutegravir-related ADRs) to raise a sufficient sample for evaluating the intervention's effect on the reporting rates of all ADRs and those specific to dolutegravir and isoniazid. A cluster size of ten health-care workers and coefficient of variation of 0·25 were assumed.^15^ To achieve a power of 80% at the 95% confidence level, 228 clusters (114 per group) were required. An adjustment was made to account for non-participation, loss to follow-up, and contamination,^13^, ^14^, ^16^ yielding 382 clusters (191 per group), with an estimated 3820 health-care workers (1910 per group). The trial anticipated 306 dolutegravir-related ADR reports, 136 and 170 from the control and intervention groups, respectively, during 10 440 000 dolutegravir person-months of observation (appendix p 7).

We present frequencies with percentages for categorical variables; and medians with IQRs, (also 10th and 90th percentiles) for scale variables due to non-normal distributions. Baseline characteristics by study group are provided. We assessed contamination by computing the proportion of control clusters that submitted app reports (appendix p 7).^14^

The study adhered to the Consolidated Standards of Reporting Trials, with an extension for cluster-randomised trials, and employed an intention-to-treat analysis (the primary outcome was analysed in all sites that received the allocated training). Per-protocol analysis was not done because contamination was minimal (deviation from protocol as per-protocol analysis was pre-planned). Before formal analysis, all ADR reports from the 367 clusters during the study period were retrieved and de-duplicated based on similarities in four key data fields: age, sex, adverse reaction, and suspect medicine. We excluded COVID-19 vaccine-related adverse events because Med Safety was not designed to capture them. We calculated the ADR reporting rate for each cluster by dividing the number of ADR reports it submitted during its follow-up by the total dolutegravir person-months of follow-up. Dolutegravir person-months were obtained by multiplying follow-up months of each cluster by the baseline number of people with HIV receiving dolutegravir-based regimens in that cluster. The cluster reporting rates were computed via mixed-effects negative binomial regression using the cluster's estimated number of dolutegravir person-months of observation as offset. The outcome for isoniazid-related ADRs was the number of ADR reports per cluster, without adjusting for isoniazid person-months. Unlike dolutegravir, which individuals received continuously, isoniazid was a short-course treatment (6–9 months) and inconsistently provided.

We evaluated the intervention's effectiveness on the primary and secondary outcomes using mixed-effects negative binomial regression with robust standard errors to allow for overdispersion of ADR counts, adjusting for clustering by incorporating random effects for health facilities nested within health facility levels. The models included randomisation group as a fixed effect and an offset for person-months of follow-up and were also fitted separately after excluding clusters with outlier non-serious dolutegravir-related ADR counts. In sensitivity analysis, to compare with results from mixed-effects negative binomial regression, multiple linear regression was done. Incidence rate ratios (IRRs) are reported with 95% CIs and were derived from mixed-effects negative binomial regression models. Intraclass correlation coefficients (ICCs) were calculated by dividing the variability between cluster levels by the sum of the variability between cluster levels and variability within clusters. ICCs were computed for all clusters and separately after excluding clusters with outlier ADR counts. For isoniazid, we compared the distribution of ADR reports between study groups using the Wilcoxon rank-sum test.

In post-hoc analysis, we assessed the intervention's durability on increasing ADR reporting by comparing its effectiveness in the first 6 months when reminders were given to reporters versus the next 6 months without reminders, spanning the initial 12 months of follow-up, using mixed-effects negative binomial regression modelling. We also compared Med Safety's effectiveness in the first 12 months versus the next 12 months, spanning the initial 24 months of follow-up. Only reports submitted within each period were included in the analysis. Modelling included only clusters with completed follow-up per period, 367 clusters in the 12-month period and 297 in the 24-month period.

To assess data quality and address potential variability in ADR report classification, two authors (RK and GI) independently reviewed a random 30% sample of serious ADR reports. Disagreements were resolved by consensus and compared with the national pharmacovigilance database (appendix p 11). To evaluate feasibility and acceptability of the intervention, we determined frequencies and percentages of the reasons for failed Med Safety installation at enrolment in the intervention group (appendix p 12). All analyses employed STATA version 15.

Following an analysis of the interim database on Sept 30, 2023, the DSMB recommended termination of the trial on Dec 12, 2023. This recommendation was based on the observation that there were three times as many ADR reports at mid-term as initially anticipated during the trial's design phase. In addition, there was a likely 50% overshoot in total person-time of observation, exceeding initial projections (appendix p 12).

### Role of the funding source

The funders of the study had no role in study design, data collection, data analysis, data interpretation, or writing of the report.

## Results

Between Aug 11, 2020, and Nov 1, 2022, 367 (96%) of 382 eligible clusters were enrolled into the trial and received the allocated intervention (184 sites in the intervention group and 183 in the control group). None of the 367 clusters were lost to follow-up. We recorded the reasons for excluding 15 clusters (figure). Overall, 3176 health-care workers were approached and 2464 (78%) consented (figure). Outcome data collection was completed on Dec 12, 2023. The median duration of cluster follow-up was 37·8 months (IQR 34·2–39·8). All 367 clusters completed at least 12 months of follow-up. Of these, 297 completed at least 24 months, 213 completed at least 30 months, and 195 completed at least 36 months. The total follow-up time was 15 564 390 dolutegravir person-months. Approximately 80% of health-care workers had access to a smartphone (table 1).

**Figure:**
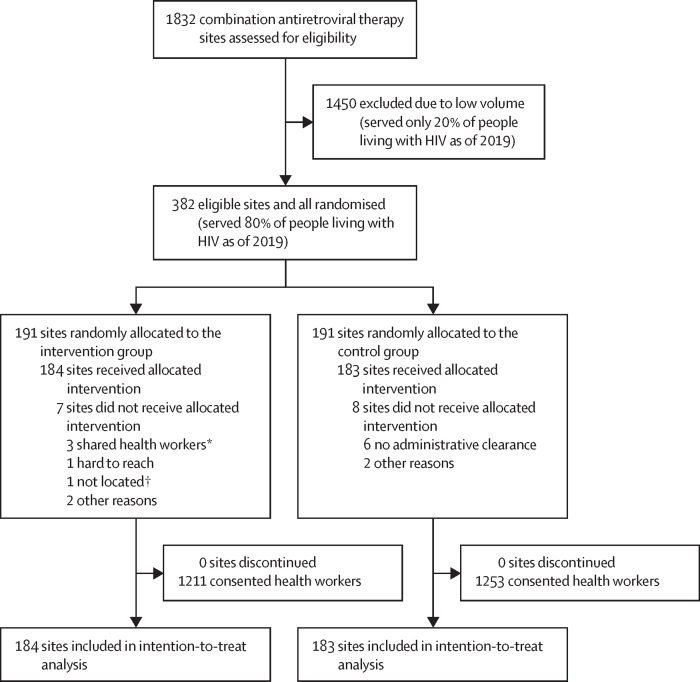
Trial profile *The same health-care workers served in multiple clusters. †This facility was included on the AIDS Control Centre list of facilities, but it could not be found when the research assistants looked for it.

**Table 1:** Baseline characteristics of the health facilities and health-care workers in Uganda

			**Control group**	**Intervention group**
Number of health facilities, n			183	184
Number of health-care workers, n			1211	1253
Health facility characteristics				
	Geographical region of Uganda, n (%)			
		Central	59 (32%)	56 (30%)
		Eastern	37 (20%)	38 (21%)
		Northern	40 (22%)	43 (23%)
		Western	47 (26%)	47 (26%)
	Facility ownership: Government, n (%)		141 (77%)	149 (81%)
	Level of health facility, n (%)			
		Health centre 2	18 (10%)	13 (7%)
		Health centre 3	52 (28%)	63 (34%)
		Health centre 4	60 (33%)	51 (28%)
		Hospital	44 (24%)	45 (24%)
		Specialised clinic	9 (5%)	12 (7%)
	Mobile telephone network available, n (%)		161 (88%)	170 (92%)
	Internet connection provided to health-care worker, n (%)		88 (48%)	96 (52%)
	Total number of health-care workers, median (IQR); 10th percentile, 90th percentile		22 (16–31);12, 56	20 (13–30);10, 59
	Number of people with HIV taking dolutegravir, median (IQR); 10th percentile, 90th percentile		900 (399–1840);178, 3100	868 (398–1648);100, 3925
	Number of people with HIV taking isoniazid preventive therapy, median (IQR); 10th percentile, 90th percentile		150 (48–900);16, 1916	176 (53–680);10, 1500
Health worker characteristics				
	Sex: proportion of health-care workers at health facility, median (IQR)			
		Male	40 (25–58)	40 (25–58)
		Female	60 (42–75)	60 (42–75)
	Highest education level: proportion of health-care workers at health facility, median (IQR)			
		Below bachelor's degree	80 (71–100)	86 (68–100)
		Bachelor's degree or higher	20 (0–29)	14 (0–33)
	Smartphone ownership: proportion of health-care workers at health facility, median (IQR)		80 (60–100)	83 (67–100)
	Age, years, median (IQR) of mean cluster ages		36 (33–39)	35 (33–39)
	Professional years of experience, median (IQR) of mean cluster years of experience		7 (6–9)	7 (5–9)
	Number of medical doctors, n (%)			
		Zero	93 (51%)	105 (57%)
		One	44 (24%)	46 (25%)
		Two or more	46 (25%)	33 (18%)
	Number of pharmacists or pharmacy technicians, n (%)			
		Zero	111 (61%)	113 (61%)
		One	39 (21%)	38 (21%)
		Two or more	33 (18%)	33 (18%)
	Number of clinical officers, n (%)			
		Zero	19 (10%)	29 (16%)
		One	60 (33%)	56 (30%)
		Two	50 (27%)	58 (32%)
		Three or more	54 (30%)	41 (22%)
	Number of midwives or nurses, median (IQR); 10th percentile, 90th percentile		4 (2–6);1, 10	4 (2–6);1, 10

During the study period, 162 (44%) of 367 clusters submitted at least one ADR report. Specifically, 89 (48%) of 184 intervention clusters and 73 (40%) of 183 control clusters submitted at least one ADR report (appendix pp 12–13). Furthermore, 118 (32%) of 367 clusters filed at least one dolutegravir-related ADR report, with 66 (36%) of 184 in the intervention group and 52 (28%) of 183 in the control group. Overall, 3622 ADR reports were submitted, with 1369 (38%) classified as serious. A total of 1343 dolutegravir-related ADR reports were submitted, with 600 (45%) classified as serious. The five most frequent dolutegravir-related serious ADRs were hyperglycaemia (239 [40%] of 600), skin reactions (62 [10%] of 600), peripheral neuropathies (54 [9%] of 600), general body weakness (48 [8%] of 600), and erectile dysfunction (45 [8%] of 600; 45 [21%] of 212 male serious dolutegravir-related ADRs). A total of 640 isoniazid-related ADR reports were submitted, with 142 (22%) classified as serious, of which the two most commonly reported were skin reactions (37 [26%] of 142) and general body weakness (27 [19%] of 142; data not shown).

For the primary outcome, the intervention group had a higher overall mean ADR reporting rate of 10·6 (SD 17·4) reports per 100 000 person-months of follow-up, compared with 5·9 (17·9) reports per 100 000 person-months in the control group (IRR 1·73 [95% CI 1·26–2·37]; p=0·001). The ICC for all ADR reports was 0·22, indicating that 22% of the variability in reporting rates was due to differences between cluster levels (table 2). The ICC was 0·32 after excluding four level 4 clusters with outlier non-serious dolutegravir-related ADR (appendix p 8).

**Table 2:** Effectiveness of the Med Safety App on the rate of ADRs reporting by health-care workers in Uganda

	Mean reporting rate (SD) per 100 000 person-months*		**Incidence rate ratio**†**(95% CI)**	**p value**	**ICC**	**Overdispersion, ln alpha (SD)**‡§	
	Control group	Intervention group				All clusters (n=367)	Clusters, no outliers (n=363)
**Primary outcome**							
All ADRs	5·9 (17·9)	10·6 (17·4)	1·73 (1·26–2·37)	0·001	0·22	0·44 (2·30)	0·70 (1·71)
**Secondary outcomes**							
Dolutegravir-related ADRs	2·2 (18·8)	4·4 (18·4)	1·92 (1·42–2·60)	<0·001	0·18	0·94 (3·06)	1·04 (3·62)
All serious ADRs	3·2 (17·9)	4·0 (17·3)	1·22 (0·83–1·78)	0·304	0·48	1·06 (4·60)	1·14 (4·19)
Serious dolutegravir-related ADRs	1·2 (19·6)	1·6 (18·6)	1·30 (0·62–2·72)	0·486	0·49	1·53 (4·60)	1·44 (4·00)
All non-serious ADRs	3·2 (19·4)	6·5 (18·7)	1·97 (1·51–2·59)	<0·001	0·14	0·50 (1·72)	0·91 (3·62)
Non-serious dolutegravir-related ADRs	1·3 (20·6)	3·4 (19·7)	2·56 (2·11–3·11)	<0·001	0·06	−15·67 (22·80)	0·43 (4·76)

ADRs=suspected adverse drug reactions. ICC=intraclass correlation coefficient measured as a proportion.

* Model-adjusted marginal means of ADR-reporting rates estimated using mixed-effects negative binomial regression.

† A mixed-effects negative binomial regression model was used to estimate the rate ratios.

‡ The cluster design was accounted for by including random intercepts at the cluster level and for individual clusters.

§ The multiplier ln alpha describes the extent of extra Poisson variation or overdispersion for all clusters and when 4 outlier-clusters with 10-times or 100-times more non-serious dolutegravir-related ADR counts are excluded.

For secondary outcomes, the mean reporting rate of dolutegravir-specific ADRs in the intervention group was 4·4 (SD 18·4) per 100 000 person-months of follow-up, higher than in the control group (2·2 [18·8]; IRR 1·92 [95% CI 1·42–2·60], p<0·001). The ICC was 0·18 for dolutegravir-related ADR reports, indicating that 18% of the variability in reporting rates was due to differences between cluster levels (table 2). The ICC was 0·30 after excluding outlier clusters (appendix p 8). The mean ADR reporting rate for all serious ADR reports per 100 000 person-months was 4·0 (SD 17·3) in the intervention group and 3·2 (17·9) in the control group, with no meaningful difference (IRR 1·22 [95% CI 0·83–1·78], p=0·304). For serious dolutegravir-related ADR reports per 100 000 person-months, the mean ADR reporting rate of 1·6 (SD 18·6) in the intervention group was not different from that of the control group (1·2 [19·6]; IRR 1·30 [95% CI 0·62–2·72], p=0·486). By contrast, for all non-serious ADR reports, the mean ADR reporting rate per 100 000 person-months was 6·5 (SD 18·7) in the intervention group and was higher than in the control group (3·2 [19·4]; IRR 1·97 [95% CI 1·51–2·59], p<0·001). For non-serious dolutegravir-related ADRs, the mean ADR reporting rate was 3·4 (19·7) in the intervention group and was higher than in the control group (1·3 [20·6]; 2·56 [95% CI 2·11–3·11], p<0·001; table 2). The ICCs were 0·14 for all non-serious ADR reports and 0·06 for dolutegravir-related non-serious ADR reports; and 0·28 and 0·17 after excluding outlier-clusters (appendix p 8). The ICCs were higher at 0·48 for all serious ADR reports and 0·49 for serious dolutegravir-related ADR reports (table 2). The mean number of isoniazid-related ADR reports in the intervention was different from the control (2·0 [SD 16·8] *vs* 1·5 [11·4]; p=0·021).

In sensitivity analysis, multiple linear regression yielded similar IRRs as the mixed-effects negative binomial regression (appendix pp 8, 10).

In post-hoc analysis, the intervention effect was observed at 24 months of follow-up (appendix p 9). In the initial 12 months of follow-up, for all ADR reports, the magnitude of IRR for the first 6 months (IRR 2·51, [95% CI 1·18–5·32], p=0·016) was similar to that for the second 6 months (IRR 2·03 [95% CI 1·09–3·76], p=0·025). In the initial 24 months of follow-up, for all ADR reports, the IRR for the first 12 months (IRR 2·72 [95% CI 1·84–4·03], p<0·001) was similar to that for the second 12 months (IRR 3·42 [95% CI 2·03–5·76], p<0·001; appendix p 9). For dolutegravir-related ADR reports, during the initial 12 months of follow-up, the IRR for the first 6 months (IRR 2·84 [95% CI 1·24–6·53], p=0·014) was similar to that for the second 12 months (IRR 1·55 [95% CI 0·72–3·36], p=0·267). In the initial 24 months of follow-up, for dolutegravir-related ADR reports, the IRR in the first 12 months (IRR 2·36 [95% CI 1·56–3·58], p<0·001) was similar to that in the second 12 months (IRR 3·42 [95% CI 2·03–5·76], p<0·001; appendix p 9). Details of data quality are reported in the appendix (p 11).

## Discussion

This pragmatic cluster-randomised, controlled trial shows that Med Safety is effective in improving the rate of ADR reporting among health-care workers in Uganda, particularly for dolutegravir-related ADRs, and was more effective for non-serious ADRs. The estimated effect sizes remained robust to the exclusion of clusters with outlier ADR counts. The positive impact of Med Safety on ADR reporting by Ugandan health-care workers highlights the value of smartphone apps in strengthening pharmacovigilance. Med Safety's success could be replicated in other LMICs, including in those where it has already been deployed.^17^ To our knowledge, this is the first large, pragmatic cluster-randomised controlled trial to test the impact of a mobile app on ADR reporting by health-care workers, and adds novel evidence from an LMIC setting. Existing evidence on the effectiveness of mobile apps for ADR reporting by health-care workers in LMICs is limited to a small cluster-randomised trial in Laos.^18^

Our findings align with previous reports from systematic reviews which found that ADR reporting rates increased with the use of other electronic systems (web-based and electronic health records).^4^, ^7^, ^16^, ^19^, ^20^, ^21^ None of the electronic systems in these systematic reviews were smartphone apps and LMICs were under-represented. In high-income countries (HICs), the widespread use of internet-connected computers enhances web-based systems and electronic health records, facilitating their adoption.^22^ In contrast, in LMICs, smartphones are more prevalent than internet-connected computers, supporting greater uptake of mobile apps.^23^ Moreover, health-care workers in LMICs are willing to use Med Safety despite high internet costs, poor connectivity, smartphone incompatibilities, complex registration requirements, difficulty recognising ADRs, and inadequate feedback to reporters.^11^

The modest increase in ADR reporting with Med Safety and web-based systems can be attributable to their passive nature, as these electronic tools provide convenience yet do not offer the active promotion seen with educational interventions.^4^ Active educational interventions, especially face-to-face engagements, tend to result in higher ADR reporting rates compared with passive electronic methods.^24^, ^25^ Trials in HICs that have evaluated active, standalone non-electronic interventions often find modest improvements in ADR reporting, similar to that seen in our Med Safety trial. An educational programme in Spain doubled ADR reporting, and reminders in Sweden increased ADR reporting by 1·5-times.^26^, ^27^ Thus, standalone interventions, whether passive or active, might have a modest impact on ADR reporting rates. By contrast, multifaceted interventions that combine passive and active strategies could be more effective, increasing the ADR reporting rate by up to 14-times.^28^ However, a recent Cochrane review in HICs indicates an increase in the ADR reporting rate by 3-times following an educational intervention combined with reminder cards and ADR forms, although with low-certainty evidence.^29^ The effectiveness of interventions (electronic and non-electronic) in promoting ADR reporting is amplified by the low pre-intervention reporting rates;^4^ however, their effect has only been sustained in the short term to medium term.^7^

Med Safety was effective at 24 months which suggests its potential to drive medium-term improvements in ADR reporting in Uganda, although its long-term impact remains uncertain. An overview of systematic reviews not involving mobile app interventions indicated that electronic tools can improve ADR reporting in the short term to medium term, although the quality of evidence was weak.^7^ In Australia, the effect of an adverse event recording module in pharmacy software diminished within 1 year, despite its initial boost to ADR reporting.^30^ Sustaining Med Safety's effectiveness in the medium term to long term will require continuous support for health-care workers through regular training, awareness programmes, and multifaceted interventions, as shown by other successful initiatives.^7^

The study found no difference in the reporting rates of serious ADRs between study groups, contrasting with the effects seen with non-serious ADRs. However, the relationship observed with serious ADRs aligns with the trends seen across all ADRs and non-serious ADRs. A potential explanation is that serious ADRs are more likely to be reported, irrespective of the pharmacovigilance method used.^31^ The impact of Med Safety on the reporting of serious dolutegravir-related ADRs, such as hyperglycaemia and erectile dysfunction, requires further investigation. In Europe, reporting serious ADRs becomes a priority after the first 2 years of intensive monitoring following a product's marketing authorisation.^31^ Consequently, non-serious ADRs, including new ones that might emerge after the intensive monitoring period, could go unreported despite their clinical relevance. Drug safety should be an integral part of patient care. Thus, to promote comprehensive monitoring, health-care workers should be encouraged to report both serious and non-serious ADRs beyond the initial 2-year intensive monitoring period. Med Safety provides an additional channel for capturing non-serious reports, thereby enriching the overall safety profile of monitored medicines. This is particularly valuable for understanding the full safety profile of new medicines, such as dolutegravir.

Although the Med Safety app contributed to increased ADR reporting, the app's impact on the quality of reporting remains to be established. Most of the serious ADR reports evaluated by independent reviewers met the WHO minimum reporting criteria, which ensures the inclusion of basic patient, suspect medicine, adverse event, and reporter information. However, these serious ADR reports frequently lacked essential clinical details such as onset date, outcome, and start date or route of administration of the suspect medicine—information that is critical for causality assessment and effective signal detection. These gaps highlight that without sufficient attention to data quality, digital reporting tools could yield large volumes of data with poor use for regulatory action. The impact of Med Safety on the quality of ADR reporting will be reported in detail elsewhere.

The estimated ICCs for all ADRs, dolutegravir-related ADRs, and non-serious ADRs suggest that most of the variability in reporting rates is driven by differences within clusters rather than between cluster levels. This suggests that interventions to improve ADR reporting should primarily target individual health-care workers within clusters, focusing on measures such as tailored training and infrastructural support to encourage reporting. For serious ADRs, variability is driven equally by cluster-level and within-cluster factors, highlighting the need for targeted interventions at both levels.

The trial was terminated after surpassing the total follow-up person-time and exceeding the initially projected number of ADR reports, which represents a strength of the trial. Other strengths include its randomised controlled design conducted in a real-world setting, the large sample size, comprehensive follow-up, and low level of contamination. Collectively, these attributes highlight the trial's internal validity and provide robust evidence of Med Safety's effectiveness. Limitations of the study include the experimental nature of the study and its specific context to Uganda, potentially limiting generalisability to other LMICs. However, challenges to ADR reporting are often similar across LMICs, and as Med Safety is adaptable, the findings are likely to be generalisable to other LMICs. After excluding outlier-clusters, the ICCs were higher, indicating the influence of extreme values on variability; however, the estimated effect sizes remained robust.

Future research should explore mechanisms for sustaining ADR reporting, factors influencing mobile app usage, patterns of reporting serious ADRs via mobile apps, and the impact of improved ADR reporting on patient outcomes and drug-safety policies. Additionally, Med Safety's scalability elsewhere, its effectiveness for other medications, and influence on health-care workers’ behaviour should be investigated.

In conclusion, Med Safety increased ADR reporting rates among health-care workers in Uganda, particularly non-serious and dolutegravir-related ADRs, compared with health-care workers not using Med Safety and the impact was seen medium-term (up to 24 months) as studied. Integrating digital technologies into pharmacovigilance systems could improve drug-safety monitoring and contribute to more robust, data-driven regulatory decision making in Uganda and other LMICs. It is, however, also important to implement multifaceted interventions including comprehensive training programmes for health-care workers to sustain high rates of ADR reporting.

### Contributors

### Data sharing

An anonymised dataset will be made available upon reasonable request to the corresponding author (kiguba@gmail.com).

## Declarations of interests

MP has received grants paid to his institutions from the Medical Research Council (MRC) Clinical Pharmacology Training Scheme (co-funded by MRC and Roche, Union Chimique Belge, Eli Lilly, and Novartis), the MRC Medicines Development Fellowship Scheme (co-funded by MRC and GSK, AstraZeneca, Optum, and Hammersmith Medicines Research), ESRC, National Institute of Health Research, NHS Genomics Unit, NHS Race and Health Observatory, Health Date Research UK, EU Innovative Medicines Initiative, and Innovate UK. He has participated on an advisory board for Bosch Health Foundation and Qatar Precision Health Initiative, and had a leadership or fiduciary role for Commission on Human Medicines, British Heart Foundation, and MRC. He has developed an HLA genotyping panel with MC Diagnostics but does not benefit financially from this. He is part of the IMI Consortium ARDAT (www.ardat.org); none of these funding sources have been used for the current research. All other authors declare no competing interests.
