# Supplementary material for: Adverse drug reaction reporting with the Med Safety app in Uganda: a cluster-randomised, controlled trial
Source: Lancet Glob Health. 2025 Sep 17;13(10):e1761–70. doi: 10.1016/S2214-109X(25)00299-2 (PMC12446902; doi:10.1016/S2214-109X(25)00299-2)
Supplement: Supplementary appendix [file mmc1.pdf]

THE LANCET
Global Health

Supplementary appendix

This appendix formed part of the original submission and has been peer reviewed. We post it as supplied by the authors.

Supplement to: Kiguba R, Ndagije HB, Mwebaza N, et al. Adverse drug reaction reporting with the Med Safety app in Uganda: a cluster-randomised, controlled trial. *Lancet Glob Health* 2025; **13:** e1761–70.

Supplementary Material

Table of Contents

Supplement 1: Study Questionnaires ... 3

Supplement 2: Expected number of adverse drug reaction reports submitted by healthcare workers .. 7

Supplement 3: Compliance with randomisation allocation ... 7

Supplement 4: Effectiveness of the Med Safety App ... 8

Supplement 5: Durability of Intervention Effectiveness ... 9

Supplement 6: Effectiveness of the Med Safety App based on alternative modelling approaches ... 10

Supplement 7: Basis of the mixed-effects negative binomial regression analysis ... 11

Supplement 8: Quality of adverse drug reaction reports ... 11

Supplement 9: Feasibility and Acceptability of the Med Safety App ... 12

Supplement 10: Monitoring of the trial ... 12

Supplement 11: Distribution of adverse drug reaction reports by health facilities that submitted at least one ADR report ... 12

Supplement 1: Study Questionnaires

Study Questionnaire for Healthcare Workers

Research Assistant: ____________________ Code: [ ]

Region: ______________________________ Code: [ ]

Health Facility Name: ____________________________ Code: [ ]

Date of Interview: ____/_____/_____

Adverse drug reaction (ADR) is an unwanted or harmful reaction experienced following the administration of a drug or combination of drugs under normal doses/conditions of use & is suspected to be related to the drug. An ADR might require the drug to be discontinued or the dose reduced.

SECTION A: DEMOGRAPHICS

1. Gender:
   [1] Male
   [2] Female

2. Age (*in completed years*): _____

3. Do you own a working smartphone?
   [1] Yes
   [2] No

3a) If ***YES*** to **Q3**, are you willing to join a WhatsApp group? [1] Yes [2] No
3b) If ***YES*** to **Q3a**, can you share your WhatsApp number? _____________

4. Do you own a simple working mobile phone *(not a smartphone)*?
   [1] Yes
   [2] No

5. Highest Education Level:
   [1] Certificate
   [2] Diploma
   [3] Bachelors
   [4] Masters
   [5] Other, (*specify*)______________

6. Professional experience (*Years*): [ ]

7. If less than 1 year in **Q6**, state number of *completed months* _____

8. Do you use any of the following? (***Tick all that apply***)
   [1] WhatsApp
   [2] Facebook
   [3] Twitter
   [4] Other, (*specify*) _____________

9. Professional Cadre:
   [1] Physician
   [2] Medical Officer
   [3] Pharmacist
   [4] Nurse/Midwife
   [5] Clinical Officer
   [6] Pharmacy Technician
   [7] Lay Counsellor
   [8] Expert Client
   [9] Other, (*specify*) _____________

10. Health Facility Type:
    [1] Public
    [2] Private Not-for-Profit
    [3] Private for-Profit

11. Health Facility Status:
    [1] Regional Referral Hospital
    [2] Hospital
    [3] Health Centre IV
    [4] Health Centre III
    [5] Health Centre II
    [6] Private Clinic
    [7] Other, (*specify*) _____________

SECTION B: DOLUTEGRAVIR-LINKED AND ISONIAZID-LINKED ADRS

Please, complete the questionnaire by indicating the appropriate responses.

1. What is the approximate number of HIV-positive patients you see per day? _____

Dolutegravir-linked adverse drug reactions

2. Dolutegravir-based antiretroviral therapy was recently rolled out in the treatment of HIV-positive patients, have you ***ever encountered*** any dolutegravir-linked adverse drug reactions (ADRs) amongst the HIV-positive patients in your care?
   [1] Yes [2] No [9] I don't know

3. In the past 4-weeks, have you ***suspected*** any dolutegravir-linked ADRs?
   [1] Yes [2] No (***Skip to 5***) [9] I don't know (***Skip to 5***)

4. If ***YES*** to **Q3**, how many cases of dolutegravir-linked ADRs have you ***suspected*** in the past 4-weeks? _____

5. In the past 4-weeks, have you ***received*** any ***patient-complaints*** of dolutegravir-linked ADRs?
   [1] Yes [2] No (***Skip to 7***) [9] I don't know (***Skip to 7***)

6. If ***YES*** to **Q5**, how many ***patient-complaints*** of dolutegravir-linked ADRs have you ***received*** in the past 4-weeks? _____

7. Briefly describe the most recent ***dolutegravir-linked*** ADR you have encountered giving details on ***patient age***, ***sex***, ***approximate ADR-date, details of the adverse drug reaction***, ***severity*** *(mild, moderate, severe)*, ***seriousness*** (*led to or prolonged hospitalization, caused incapacitation, led to any other medically significant condition or death*), ***clinical outcome*** & ***action taken***:
   Patient age [ ] years; **sex** [M or F]; **approximate ADR-date** [*mm/yyyy*: __ / ____]

   ADR description (*include severity, seriousness, clinical outcome and action taken*)____
   ____
   ____

Isoniazid preventive therapy-linked adverse drug reactions

8. HIV-positive patients are at a higher risk of dying when they develop active tuberculosis (TB). During the past one year, Ministry of Health scaled up the roll-out of isoniazid preventive therapy (IPT) among HIV-positive patients to significantly reduce these patients' risk of developing active TB. IPT, however, has been associated with ADRs. Have you ***ever encountered*** any IPT-linked ADRs amongst the HIV-positive patients in your care?
   [1] Yes [2] No [9] I don't know
9. In the past 4-weeks, have you ***suspected*** any isoniazid preventive therapy-linked ADRs?
   [1] Yes [2] No (***Skip to 11***) [9] I don't know (***Skip to 11***)

10. If ***YES*** to **Q9**, how many isoniazid preventive therapy-linked ADRs have you ***suspected*** in the *past 4-weeks*? _____

11. In the past 4-weeks**,** have you ***received*** any ***patient-complaints*** of isoniazid preventive therapy-linked ADRs?

    [1] Yes [2] No (***Skip to 13***) [9] I don't know (***Skip to 13***)

12. If ***YES*** to **Q11**, how many ***patient-complaints*** of isoniazid preventive therapy-linked ADRs have you ***received*** in the past 4-weeks? _____

13. Briefly describe the most recent ***isoniazid-linked*** ADR you have encountered giving details on ***patient age***, ***sex***, ***approximate ADR-date, details of the adverse drug reaction***, ***severity*** *(mild, moderate, severe)*, ***seriousness*** (*led to or prolonged hospitalization, caused incapacitation, led to any other medically significant condition or death*), ***clinical outcome*** & ***action taken***:
    Patient age [ ] years; **sex** [M or F]; **approximate ADR-date** [*mm/yyyy*: __ / ____]

    ADR description (*include severity, seriousness, clinical outcome and action taken*)
    ____
    ____
    ____

14. In the past 6-months, have you ***reported*** any suspected ADRs experienced by the HIV-positive patients under your care?
    [1] Yes [2] No (***Skip to Q23***) [9] I don't know (***Skip to Q23***)

15. If ***YES*** to **Q14**, how many ***ADR reports*** did you make? [ ][ ]

16. If ***YES*** to **Q14**, which drug classes were suspected? (***Tick all that apply***)
    [1] Antiretroviral therapy
    [2] Isoniazid preventive therapy
    [3] Antituberculosis therapy
    [4] Other, (*specify*)____

17. If ***YES*** to **Q14**, which of the following drugs were implicated? (***Tick all that apply and mention number of reported ADRs in past 4-weeks for options [1] & [2]***)
    [1] Dolutegravir: *Number of ADRs **reported** in the past 4-weeks* _____
    [2] Isoniazid: *Number of ADRs **reported** in the past 4-weeks* _____
    [3] Other, (*specify*) ______________

18. If ***YES*** to **Q14**, to whom did you report the most recent ADR? (***Tick all that apply***)
    [1] District Health Officer
    [2] Health Management Information System
    [3] Immediate Supervisor
    [4] National Drug Authority - National Pharmacovigilance Center
    [5] Other, (*specify*)__________________

19. If ***YES*** to **Q14**, how did you report the most recent suspected ADR? (***Tick all that apply***)
    [1] Verbally
    [2] Written paper report
    [3] Written online/website report
    [4] Other, (*specify*) __________________

20. If ***YES*** to **Q14**, did you get feedback on the ADR(s) you reported?
    [1] Yes [2] No [9] Not sure

21. If ***YES*** to **Q20**, what form of feedback?
    ____

22. In special situations, the National Drug Authority may need additional information about reported ADRs. Would you be willing to answer further questions after sending a report?
    [1] Yes [2] No [9] I don't know

Herbal Medicine Use

23. In the past 6-months, have you suspected that some of your patients use herbal medicines together with ARVs?
    [1] Yes [2] No

24. If YES to **Q23**, have you inquired from the patients whether they use herbal medicines together with ARVs?
    [1] Yes [2] No

25. In the past six months, have any of your patients reported to you that they use herbal medicines together with ARVs? [1] Yes [2] No

26. Have any of your patients presented with ADRs likely to be linked to the use of herbal medicines?
    [1] Yes [2] No

We appreciate the time you've taken to respond to this survey. Thank you!

Study Questionnaire for Health Facility Data

Research Assistant: ____________________ Code: [ ]

Region: ______________________________ Code: [ ]

Health Facility Name: ____________________________ Code: [ ]

Date of Enrolment of Health Facility: ____/_____/_____

Average daily number of ART patient visits at the Health Facility: [ ][ ][ ]

Total number of patients on dolutegravir-containing antiretroviral therapy [ ][ ][ ][ ]

Total Number of patients on Isoniazid Preventive Therapy [ ][ ][ ][ ]

Number of healthcare professionals at the Health Facility:

- Medical Doctors [ ][ ]
- Pharmacists [ ][ ]
- Clinical Officers [ ][ ]
- Nurses/Midwives [ ][ ]
- Pharmacy Technicians [ ][ ]
- Village Health Teams [ ][ ]
- Lay Counsellors [ ][ ]
- Expert Clients [ ][ ]
- Other (*specify*) ………………………. [ ][ ]

Does the Health Facility provide health workers with internet access?

1. No 2. Yes 9. Unknown

If YES, describe the form of internet access provided (*wireless, wired, data etc.*):

____

____

Is mobile telephone network connectivity available at the Health Facility?

1. No 2. Yes 9. Unknown

If YES, describe the reliability of mobile telephone network connectivity:

____

____

Supplement 2: Expected number of adverse drug reaction reports submitted by healthcare workers

Table S1: Expected number of adverse drug reaction reports during 30 months of follow-up		
**Outcome**	**Control arm**	**Intervention arm**
**Assumption 1:** 382 admissible sentinel sites look after 348,000 dolutegravir (DTG)-treated patients and 300,000 Isoniazid Preventive Therapy (IPT)-treated patients.		
Randomized DTG patient-load	174,000 DTGs	174,000 DTGs
Randomized IPT patient-load	150,000 IPTs	150,000 IPTs
DTG patient-months (pms) of follow-up in 30 months post-randomisation	5,220,000 DTG-pms	5,220,000 DTG-pms
IPT patient-months (pms) of follow-up in 30 months post-randomization	4,500,000 IPT-pms	4,500,000 IPT-pms
Expected de-duplicated ADR report-rate per 100,000 DTG-pms	2.6	3.25
Expected de-duplicated ADR report-rate per 100,000 IPT-pms	1.0	1.25
Expected number of de-duplicated ADR-reports in 5,220,000 DTG-pms	**136**	**170**
Expected number of de-duplicated ADR-reports in 4,500,000 IPT-pms	**45**	**56.25**
**TOTAL expected de-duplicated ADR reports in 30 months**	**181**	**226.25**
Power: before clustering heeded	*80% (at 5% significance) assuming Poisson variation*	
ADR is suspected adverse drug reaction,		

Supplement 3: Compliance with randomisation allocation

We determined the extent of contamination based on the proportion of control sites that submitted mobile app-reports (8%, 6/73) and the proportion of ADR-reports in the control arm submitted via the mobile app (10%, 124/1224). The extent of contamination by number of mobile app-reports from control sites (8%) is well within the 30% limit set for the trial during sample size planning.[1] Note that 109 (88%) of the 124 mobile app-reports from control sites were submitted in the final 12-months of follow-up of the trial. Furthermore, 91 (83%) of these 109 mobile app-reports from control sites were submitted by a single health-facility.

Supplement 4: Effectiveness of the Med Safety App

The observation of a negative natural log-transformed overdispersion parameter (ln alpha) for *non-serious dolutegravir-related* ADR-reporting rates, indicating under-dispersion, prompted further investigation. We identified the level IV health-facility stratum as the source of the anomaly, with four health facilities exhibiting outlying *non-serious dolutegravir-related* ADR-counts (**Tables S2 & S3**).

Table S2: Effectiveness of the Med Safety App on the rate of suspected adverse drug reaction reporting by healthcare workers at 367 health facilities in Uganda

	**Incidence Rate Ratio**[a] (95% CI)	**p-value**	**ICC**[b]	**ln alpha (SD)**[c]
Primary outcome				
All ADRs	1.73 (1.26, 2.37)	0.001	0.22	0.44 (2.30)
Secondary outcomes				
Dolutegravir-related ADRs	1.92 (1.42, 2.60)	<0.001	0.18	0.94 (3.06)
All serious ADRs	1.22 (0.83, 1.78)	0.304	0.48	1.06 (4.60)
Serious Dolutegravir-related ADRs	1.30 (0.62, 2.72)	0.486	0.49	1.53 (4.60)
All non-serious ADRs	1.97 (1.51, 2.59)	<0.001	0.14	0.50 (1.72)
Non-serious Dolutegravir-related ADRs	2.56 (2.11, 3.11)	<0.001	0.06	-15.67 (22.8)

Table S3: Effectiveness of the Med Safety App on the rate of suspected adverse drug reaction reporting by healthcare workers at 363 health facilities after excluding 4 health facilities with outlier non-serious dolutegravir-related ADR counts in Uganda

	**Incidence Rate Ratio**[a] (95% CI)	**p-value**	**ICC**[b]	**ln alpha (SD)**[c]
Primary outcomes				
All ADRs	1.67 (1.20, 2.32)	0.002	0.32	0.70 (1.71)
Secondary outcomes				
Dolutegravir-related ADRs	1.87 (1.33, 2.63)	<0.001	0.30	1.04 (3.62)
All serious ADRs	1.22 (0.83, 1.79)	0.304	0.58	1.14 (4.19)
Serious Dolutegravir-related ADRs	1.45 (0.88, 2.37)	0.143	0.47	1.44 (4.00)
All non-serious ADRs	1.90 (1.43, 2.53)	<0.001	0.28	0.91 (3.62)
Non-serious Dolutegravir-related ADRs	2.48 (1.96, 3.14)	<0.001	0.17	0.43 (4.76)

Supplement 5: Durability of Intervention Effectiveness

Table S4: Durability of the intervention on the rate of suspected adverse drug reaction reporting by healthcare workers in Uganda

	**Trial arm** *Average Reporting Rate (SD) per 100,000 person-months*		**Incidence Rate Ratio [a] (95% CI)**	**p-value**	**ICC [b]**	**ln alpha (SD)**
	Comparison	Intervention				
**All ADRs [c]**						
First 12-month period (n = 367)						
First 6-months	1.2 (23.7)	3.1 (21.3)	2.51 (1.18, 5.32)	0.016	0.07	1.62 (3.83)
Second 6-months	0.6 (22.5)	1.2 (21.4)	2.03 (1.09, 3.76)	0.025	0.32	1.71 (3.64)
First 24-month period (n = 297)						
First 12-months	1.9 (20.3)	5.4 (19.5)	2.72 (1.84, 4.03)	<0.001	0.08	1.19 (6.20)
Second 12-months	1.3 (24.8)	4.5 (21.8)	3.42 (2.03, 5.76)	<0.001	0.15	1.01 (2.59)
**Dolutegravir ADRs [c]**						
First 12-month period (n = 367)						
First 6-months	0.7 (27.2)	2.1 (22.8)	2.84 (1.24, 6.53)	0.014	0.00	1.33 (3.83)
Second 6-months	0.5 (28.0)	0.7 (27.4)	1.55 (0.72, 3.36)	0.267	0.15	1.80 (9.96)
First 24-month period (n = 297)						
First 12-months	1.3 (21.6)	3.2 (20.6)	2.36 (1.56, 3.58)	<0.001	0.02	0.91 (8.63)
Second 12-months	0.5 (28.5)	2.7 (24.8)	4.78 (1.61, 14.24)	0.005	0.28	1.60 (5.69)

Note: Data are mean (SD) for reporting rate, rate ratios (95% CI), proportion for ICC and mean (SD) for overdispersion, ln alpha

a The cluster design was accounted for using random intercepts at the cluster level (i.e., level of healthcare services provided) and individual clusters (i.e., specific health facility) for all estimates

a A mixed-effects negative binomial regression model was used to estimate the rate ratios

a CI is confidence interval

b ICC is Intra-class correlation coefficient

c ADR is suspected adverse drug reaction; Only the study sites that completed the 12-month or 24-month periods were included in the analysis.

Supplement 6: Effectiveness of the Med Safety App based on alternative modelling approaches

For all ADRs and dolutegravir-specific ADRs, we compared the median cluster-level reporting rates between the arms using the Wilcoxon rank-sum test. Due to the large number of clusters with zero reports, we added a constant of 0.1 to the number of ADR-reports per cluster to improve the comparisons. This adjustment allowed differentiation between clusters with zero reports based on population size. In sensitivity analysis, to compare with the results from mixed-effects negative binomial regression for the primary and secondary outcomes, we conducted multiple linear regression on the natural logarithm of the transformed cluster-level reporting rate [((cluster's ADR-count + 0.1)/dolutegravir person-months)*100,000], using randomisation arm and cluster-level as covariates, see **Table S5**. We also conducted generalised linear mixed modelling with randomisation arm as fixed-effect and cluster-level as random-effect. Both modelling approaches gave similar results to those from mixed-effects negative binomial regression. We computed the incidence rate ratios (IRRs) by dividing the ADR-reporting rates in the intervention arm by rates in the control arm, and reported the IRRs with their 95% confidence intervals (CIs). The intraclass correlation coefficient (ICC) was calculated by dividing variability between cluster levels by total variability (sum of variability between cluster-levels and variability within clusters). For Isoniazid Preventive Therapy, we compared the distribution of the number of ADR-reports between the intervention and control arms using the Wilcoxon rank-sum test.

The mean (SD) number of *isoniazid-related* ADR-reports was 2.0 (16.8) in the intervention and 1.5 (11.4) in the control arm. Although both groups had a median of 3 reports, the Wilcoxon rank-sum test showed a significant difference in the distribution of reports between the two groups (p=0.021).

Table S5: Effectiveness of the Med Safety App on the rate of ADR Reporting by healthcare workers in Uganda

	**Incidence Rate Ratio** [a] (95% CI)	**p-value**
Primary outcomes [b]		
All ADRs	1.66 (1.11, 2.48)	0.013
Secondary outcomes [b]		
Dolutegravir-related ADRs	1.82 (1.15, 2.90)	0.011
All serious ADRs	1.60 (0.47, 5.51)	0.455
Serious Dolutegravir-related ADRs	2.12 (0.67, 6.67)	0.199
All non-serious ADRs	3.72 (1.06, 13.08)	0.041
Non-serious Dolutegravir-related ADRs	4.02 (1.31, 12.37)	0.015

Note: Data are ratios with their 95% confidence intervals
a A multiple linear regression model with randomisation arm and cluster-level as covariates were used for the estimates. The outcome is ln [((# ADR-reports + 0.1)/Dolutegravir person-months)*100,000]

Supplement 7: Basis of the mixed-effects negative binomial regression analysis

The equation for the mixed-effects negative binomial regression modelling is:

ln (λij) = β0 + β1(Study Armij) + ln (dolutegravir person-months of follow-upij) + uj + vij

Where:

- i represents the individual health facility (cluster)
- j represents the cluster-level (level of healthcare services provided in a health facility)
- λij is the outcome - the expected number of ADR-reports per 100,000 dolutegravir person-months of follow-up per cluster i within cluster-level j. It is obtained after exponentiating the overdispersion parameter, **ln (λij)** in the equation
- β0 is the intercept or baseline incidence rate (conditional on zero random-effects)
- β1 is the coefficient for **Study Armij** (study arm), which represents the intervention effect
- Study Armij is the value of the intervention for cluster i within cluster-level j
- ln (dolutegravir person-months of follow-up) is the offset for the exposure time for each cluster.
- uj is the random-effect at the **cluster-level j**, following a normal distribution with mean 0 and variance σ2u
- vij is the random-effect at the **cluster** i within cluster-level j, following a normal distribution with mean 0 and variance σ2v

The random-effects uj and vij are assumed to be independent of each other and of the covariates. They capture the unexplained variability at cluster-level and for each individual cluster, respectively.

The model estimates incidence rate ratio of the intervention effect - by exponentiating coefficient β1.

Supplement 8: Quality of adverse drug reaction reports

All ADR-data underwent routine data quality checks, were processed, and securely stored by the National Pharmacovigilance Centre. Paper-forms were manually entered into the database by authorized National Pharmacovigilance Centre staff while the online and mobile app data were electronically transmitted into VigiFlow. The reports were centrally analysed at the National Pharmacovigilance Centre before their transfer into VigiBase. The pharmacovigilance manager at National Pharmacovigilance Centre anonymised the safety data by replacing the reporters' identity with unique identifiers, thereby delinking the data from the reporters' identity. Prior to data anonymisation, a drug-regulatory pharmacovigilance officer, independent of the routine assessment of ADR-data, coded reports according to the trial arm of origin. Electronic data were password-protected and utilized solely for research.

At Uganda's National Drug Regulatory Authority, trained pharmacovigilance officers classify suspected ADRs as serious or non-serious according to WHO criteria.[2] To address potential variability in this classification, two authors (RK, IG) independently reviewed a random 30% sample (411 of 1,369) of serious ADR-reports. Disagreements were resolved by consensus and compared with the national database. A 5% threshold (22 ADRs) was set for triggering a re-review of all reports; since only 18 discrepancies (4%) were identified, a re-review was unnecessary. We also evaluated the data quality of 411 random serious ADR-reports using the Kiguba criteria for comprehensive reporting and WHO criteria for the minimum required details in ADR-reports.[3]

We reviewed the quality of data in the random 411 serious ADR-reports based on five descriptors of comprehensive reporting by Kiguba et al, i.e., adverse event, suspect-medicine, route of drug administration, patient age, and ADR severity.[3] The detail in 42% (173/411) of ADR-reports was comprehensive, that is, the report had at least four of the five descriptors mentioned, although all of the reports had missing data on route of drug administration, which could otherwise be deduced from the name of the suspect medicine.

We applied the WHO criteria for the minimum detail required in submitted ADR-reports, i.e., identifiable patient (*age, sex*), adverse event (*reaction, date of onset, severity, seriousness, outcome*), suspect-medicine (*drug name, route of administration, date drug started*), and reporter (*designation, email/telephone contact, health facility*).[4] Missing data were not common for patient details [*age (7%, 29/411), sex (2%, 7/411)*]; adverse event [*reaction (3%, 11/411)*]; drug details [*suspect drug (0%, 1/411)*]; reporter details [*designation (1%, 3/411)]; health facility (0%, n=2)*], and mode of reporting [(1%, 3/411)]. All 411 ADR-reports had 100% missing data on some attributes of the adverse event (ADR-onset, ADR-outcome) and some attributes of the suspect-medicine (route of administration, start-date of administration). The reporter's contact details (telephone number, email address) were missing for 22% (91/411) of ADR-reports, implying that the NPC would not be able to access these reporters if additional information was needed.

Supplement 9: Feasibility and Acceptability of the Med Safety App

Table S6: Reasons for failure to install Med Safety in the intervention arm		
**Reason**	**Frequency**	**Percentage**
Outright refusal	3	1.5
Incompatible phones	71	35.7
Faulty smartphones	5	2.5
Poor Internet connectivity	36	18.1
No space for new apps	33	16.6
No/low battery charge on the phone	14	7.0
Busy health worker	8	4.0
Phone restriction in prison center	1	0.5
Forgot/Left phone at home	28	14.1
Total number of HWs who did not install Med Safety	199	100.0
Number of HWs who owned smartphones in intervention arm	1054	
Number of HWs who consented to the study in intervention arm	1253	

Proportion of HWs in the intervention who had smartphones	=	84% (1054/1253)
Proportion of HWs with smartphones who did not install app	=	19% (199/1054)
Proportion of HWs in the intervention who did not install app	=	16% (199/1253)
HW is healthcare worker		

Supplement 10: Monitoring of the trial

Monitoring and Evaluation Mechanism: The Research Team monitored project performance, reporting progress to the Trial Steering Committee, which provided independent evaluation based on 12-monthly reports. The HFs followed established protocols to manage PLHIV with suspected ADRs. The DSMB planned to conduct formal mid-term interim analysis to determine if the app performed substantially better than a priori estimates.[5]

Stopping rules: The trial was overseen by an independent Data and Safety Monitoring Board (DSMB). Based on historical data from the national database, the study expected 136 de-duplicated dolutegravir-associated ADR reports over 5,220,000 person-months, which corresponds to 2.6 reports per 100,000 person-months, with a target effect size reflecting a 25% increase to ~3.3 reports per 100,000 person-months. At the interim analysis, the number of ADR reports exceeded 1,000. By 12 November 2023, the follow-up period reached ~15,108,000 person-months—45% more than the initial expectation of 10,440,000 person-months—and was projected to reach ~15,600,000 person-months by 12 December 2023, a 50% increase that accounted for clustering effects. During the DSMB meeting on 20 November 2023, both the follow-up projection and the higher-than-expected ADR reports were reviewed, leading to a recommendation for trial termination.

Supplement 11: Distribution of adverse drug reaction reports by health facilities that submitted at least one ADR report

Table S7: Pattern of ADR reporting by health facilities			
**Number of reports**	**Total, n=162**	**Control, n=73***	**Intervention, n=89***
1	54	27	27
2	19	7	12
3	12	5	7
4	10	4	6
5	10	3	7
6	6	3	3

7	2		2
8	2	1	1
9	5	2	3
11	4	1	3
12	2	1	1
13	1	1	
14	6	5	1
15	1	1	
16	1		1
20	1	1	
21	1		1
22	1	1	
23	2	1	1
25	1	1	
26	1	1	
33	2	1	1
35	1	1	
38	1	1	
41	1		1
53	1		1
59	1		1
61	1		1
71	1		1
73	1		1
75	1	1	
115	1		1
171	1	1	
172	1	1	
195	1		1
206	1		1
215	1		1
333	1	1	
351	1		1
607	1		1
Mean (SD)$^{+}$	9.9 (46.9)	6.7 (31.1)	13.0 (58.5)
SE	3.7	3.6	6.2
10th percentile	0	0	0
Median	0	0	0
90th percentile	12	13	11

**44% (162/367) of sites submitted at least one ADR-report to the NPC, with 48% (89/184) of sites in the intervention and 40% (73/183) in the control.*
+The means reported here are different from those estimated by modelling because dolutegravir person-months are ignored in the estimates reported here. Also, health facilities with zero ADR reports are ignored. However, the 10th percentiles, medians and 90th percentiles reported here include all 367 sites.

Supplement 12: CONSORT 2025 checklist

Section/topic	No	CONSORT 2025 checklist item description	Reported on page no.
**Title and abstract**			
Title and structured abstract	1a	Identification as a randomised trial	1
	1b	Structured summary of the trial design, methods, results, and conclusions	2
**Open science**			
Trial registration	2	Name of trial registry, identifying number (with URL) and date of registration	2, 5
Protocol and statistical analysis plan	3	Where the trial protocol and statistical analysis plan can be accessed	5
Data sharing	4	Where and how the individual de-identified participant data (including data dictionary), statistical code and any other materials can be accessed	12
Funding and conflicts of interest	5a	Sources of funding and other support (eg, supply of drugs), and role of funders in the design, conduct, analysis and reporting of the trial	2, 8, 12
	5b	Financial and other conflicts of interest of the manuscript authors	12
**Introduction**			
Background and rationale	6	Scientific background and rationale	4
Objectives	7	Specific objectives related to benefits and harms	4
**Methods**			
Patient and public involvement	8	Details of patient or public involvement in the design, conduct and reporting of the trial	5
Trial design	9	Description of trial design including type of trial (eg, parallel group, crossover), allocation ratio, and framework (eg, superiority, equivalence, non-inferiority, exploratory)	5
Changes to trial protocol	10	Important changes to the trial after it commenced including any outcomes or analyses that were not prespecified, with reason	5
Trial setting	11	Settings (eg, community, hospital) and locations (eg, countries, sites) where the trial was conducted	5
Eligibility criteria	12a	Eligibility criteria for participants	5
	12b	If applicable, eligibility criteria for sites and for individuals delivering the interventions (eg, surgeons, physiotherapists)	5
Intervention and comparator	13	Intervention and comparator with sufficient details to allow replication. If relevant, where additional materials describing the intervention and comparator (eg, intervention manual) can be accessed	5
Outcomes	14	Prespecified primary and secondary outcomes, including the specific measurement variable (eg, systolic blood pressure), analysis metric (eg, change from baseline, final value, time to event), method of aggregation (eg, median, proportion), and time point for each outcome	6
Harms	15	How harms were defined and assessed (eg, systematically, non-systematically)	Not applicable

Sample size	16a	How sample size was determined, including all assumptions supporting the sample size calculation	6
	16b	Explanation of any interim analyses and stopping guidelines	8
Randomisation:			
Sequence generation	17a	Who generated the random allocation sequence and the method used	5
	17b	Type of randomisation and details of any restriction (eg, stratification, blocking and block size)	5
Allocation concealment mechanism	18	Mechanism used to implement the random allocation sequence (eg, central computer/telephone; sequentially numbered, opaque, sealed containers), describing any steps to conceal the sequence until interventions were assigned	5
Implementation	19	Whether the personnel who enrolled and those who assigned participants to the interventions had access to the random allocation sequence	5, 6
Blinding	20a	Who was blinded after assignment to interventions (eg, participants, care providers, outcome assessors, data analysts)	5, 6
	20b	If blinded, how blinding was achieved and description of the similarity of interventions	6
Statistical methods	21a	Statistical methods used to compare groups for primary and secondary outcomes, including harms	7
	21b	Definition of who is included in each analysis (eg, all randomised participants), and in which group	6, 7, 8
	21c	How missing data were handled in the analysis	Not applicable
	21d	Methods for any additional analyses (eg, subgroup and sensitivity analyses), distinguishing prespecified from post hoc	7
**Results**			
Participant flow, including flow diagram	22a	For each group, the numbers of participants who were randomly assigned, received intended intervention, and were analysed for the primary outcome	8
	22b	For each group, losses and exclusions after randomisation, together with reasons	8
Recruitment	23a	Dates defining the periods of recruitment and follow-up for outcomes of benefits and harms	8
	23b	If relevant, why the trial ended or was stopped	8
Intervention and comparator delivery	24a	Intervention and comparator as they were actually administered (eg, where appropriate, who delivered the intervention/comparator, how participants adhered, whether they were delivered as intended (fidelity))	8
	24b	Concomitant care received during the trial for each group	Not applicable
Baseline data	25	A table showing baseline demographic and clinical characteristics for each group	8
Numbers analysed, outcomes and estimation	26	For each primary and secondary outcome, by group:<br>● the number of participants included in the analysis	

		● the number of participants with available data at the outcome time point<br>● result for each group, and the estimated effect size and its precision (such as 95% confidence interval)<br>● for binary outcomes, presentation of both absolute and relative effect size	8, 9
Harms	27	All harms or unintended events in each group	Not applicable
Ancillary analyses	28	Any other analyses performed, including subgroup and sensitivity analyses, distinguishing pre-specified from post hoc	9
**Discussion**			
Interpretation	29	Interpretation consistent with results, balancing benefits and harms, and considering other relevant evidence	9, 10, 11
Limitations	30	Trial limitations, addressing sources of potential bias, imprecision, generalisability, and, if relevant, multiplicity of analyses	11

References

1. Torgerson DJ. Contamination in trials: is cluster randomisation the answer? *Bmj* 2001; **322**(7282): 355-7.
2. Uppsala Monitoring Centre. Glossary. 2024. https://who-umc.org/pharmacovigilance-communications/glossary/ (accessed 10 November 2024).
3. Kiguba R, Karamagi C, Waako P, Ndagije HB, Bird SM. Rare, serious, and comprehensively described suspected adverse drug reactions reported by surveyed healthcare professionals in Uganda. *PLoS One* 2015; **10**(4): e0123974.
4. Uppsala Monitoring Centre. Individual Case Safety Reports and VigiBase – the vital importance of quality. 2012. https://who-umc.org/media/163807/vigibase-the-vital-importance-of-quality-2017.pdf (accessed 10 November 2024).
5. Kiguba R, Mwebaza N, Ssenyonga R, et al. Effectiveness of the Med Safety mobile application in improving adverse drug reaction reporting by healthcare professionals in Uganda: a protocol for a pragmatic cluster-randomised controlled trial. *BMJ Open* 2022; **12**(7): e061725.
6. Hopewell S, Chan AW, Collins GS, Hróbjartsson A, Moher D, Schulz KF, et al. CONSORT 2025 Statement: updated guideline for reporting randomised trials. BMJ. 2025; 388:e081123. https://dx.doi.org/10.1136/bmj-2024-081123
